# Supplementary material for: Saccharomyces boulardii (CNCM I-745) improves intestinal damage in sepsis by remodeling intestinal flora structure
Source: Front Cell Infect Microbiol. 2025 Jul 31;15:1602792. doi: 10.3389/fcimb.2025.1602792 (PMC12350329; doi:10.3389/fcimb.2025.1602792)

Unweighted−Unifrac Anosim

P=0.021; R=0.1156

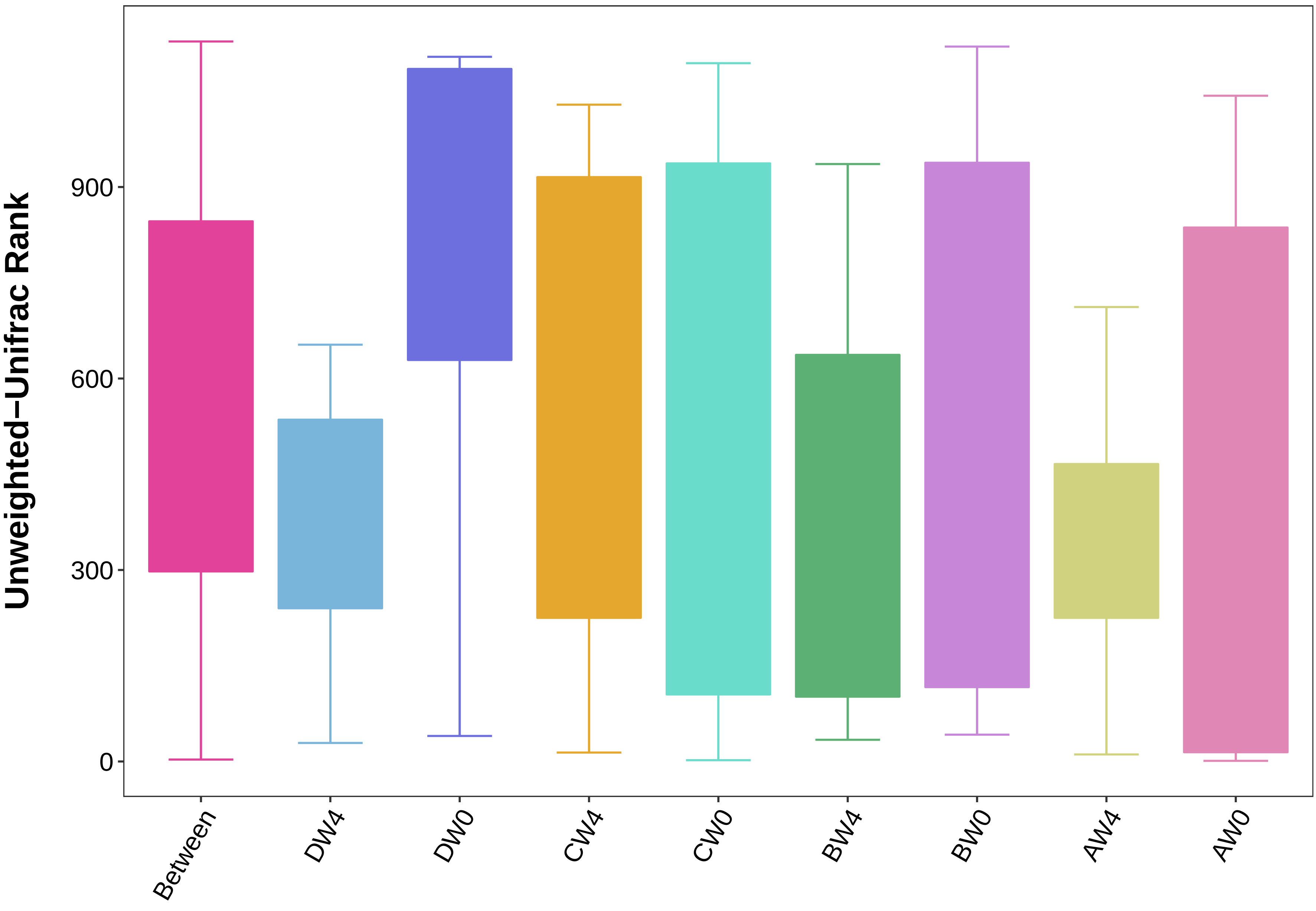

Supplement: Supplementary file 1 [file DataSheet1.zip › summary/summary/5_Beta_diversity/6_anosim_result/Beta_Anosim.unweighted_unifrac.boxplots.pdf]
